# Identification of Short Amino Acid Sequences That Correlate with Cytoplasmic Retention of Human Proteins

**DOI:** 10.3390/cells15020133

**Published:** 2026-01-12

**Authors:** Jay C. Brown, Baomin Wang

**Affiliations:** 1 Department of Microbiology, Immunology and Cancer Biology, University of Virginia School of Medicine, Charlottesville, VA 22908, USA; 2 University of Virginia School of Medicine, Charlottesville, VA 22908, USA; bw8yv@virginia.edu

**Keywords:** cytoplasmic retention sequence, bioinformatics, protein isoform

## Abstract

**Highlights:**

**What are the main findings?**

- Presented here is a novel method to identify cytoplasmic retention amino acid sequences within the sequence of human proteins.
- The new method is used to identify fifteen candidate cytoplasmic retention sequences in the human genome.

**What is the implication of the main finding?**

- Cytoplasmic retention sequences were found to be located at the same site in all isoforms of a given protein regardless of the sequence of the retention sequence itself.

**Abstract:**

One group of human proteins found in the cytoplasm, but not in the nucleus, is characterized by the presence of short (6–9 aa), specific amino acid sequences thought to be involved in retaining proteins in the cytoplasm (cytoplasmic retention sequences). While strong evidence supports the ability of some peptides to act in this way, the number of such supported cases is small. We have taken the view that the situation would be improved by enhancing the methods available to identify cytoplasmic retention (CR) sequences. Here, we describe an appropriate bioinformatic method to identify CR peptides using information about their location at the ends of cytoplasmic proteins. The method was then used to link seven different human cytoplasmic proteins with sequences suggested to have cytoplasmic retention activity. Further bioinformatic analysis was carried out with isoforms of the cytoplasmic proteins identified. Amino acid sequence information showed that while the proposed CR amino acid sequences can be the same or distinct in different protein isoforms, they are always located at the same site in the protein. For instance, while the proposed retention sequence of CCDC57 isoform X18 is MLARLVSNS, in isoform 7 it is SEPALNEL, yet the two sequences are each located between amino acids 5 and 13 in the CCDC57 sequence. The results support the view that the protein isoform is involved in determining the location of the CR sequence in a protein, while the amino acid sequence itself affects other variables such as the sub-region of the cytoplasm the protein needs to occupy. Overall, the study yielded identification of 15 candidate CR peptides in which 10 of the 15 have unrelated amino acid sequences.

## 1. Introduction

For cell biologists, it is of the utmost importance to understand factors that influence the subcellular location of proteins. A newly synthesized protein must first be trafficked to the nucleus or the cytoplasm and thereafter to the appropriate sub-region such as the endoplasmic reticulum or mitochondria. Highly specific signals must be involved at each step, and if a protein needs to change its location because of developmental or environmental events, then additional signals and interpretation of signals must be involved. Much of the cell’s machinery must be devoted to delivering its proteins to the right places, and researchers have made it a high priority to clarify the factors involved.

A landmark in the field occurred in the early 1980s when highly specific amino acid sequences were found to be able to direct proteins to the nucleus [1,2]. Identification of similar features that influence localization to the cytoplasm, however, are complicated by the fact that proteins are synthesized in the cytoplasm, making their transport to the cytoplasm unnecessary. Nevertheless, important advances have been made in identifying features that prevent proteins from leaving the cytoplasm: the cytoplasmic retention signals. For instance, a four-amino-acid sequence at the C-terminus of grp78 (HSPA5) has been found to mediate endoplasmic reticulum retention localization in COS cells [3]. Studies with fusion proteins involving a reporter protein linked to a candidate cytoplasmic retention sequence have been a rich source of information about the identity of cytoplasmic retention sequences [4,5,6,7]. Ankyrin repeats in NF-κB have been demonstrated to have cytoplasmic retention function [8].

We have taken the view that it would be an advancement if researchers had access to a rapid method to identify cytoplasmic retention protein sequences. This could be used as a first step in identifying protein–protein interactions that underlie cytoplasmic structure and function. Here, we describe a bioinformatic method designed to identify amino acid sequences able to confer cytoplasmic retention (CR) function on their home protein. Short amino acid sequences proposed for analysis were first tested for their presence in human proteins using NCBI-BLAST. Positive proteins were then screened further for the presence of the sequence at the C- or N-terminus and for protein location in the cytoplasm. Sequences with all three properties are suggested to have CR activity.

The above method was then used to test the amino acid sequence MLPRLVLNS for CR activity in human proteins, and three candidate proteins were identified. The same analysis was performed with a second candidate sequence and with isoforms of all positive proteins. The experimental pathway resulted in the identification of fifteen novel candidate CR sequences.

## 2. Materials and Methods

### 2.1. Short Amino Acid Sequences Chosen for Analysis

Initial studies were carried out with two short sequences, MLPRLVLNS and MLARLVSNS. The two were selected because preliminary analysis of short sequences (6–9 amino acids in length) demonstrated the relative abundance of MLPRLVLNS and MLARLVSNS, compared with other short sequences, among human proteins.

### 2.2. Blast Analysis

NCBI-BLAST (https://blast.ncbi.nlm.nih.gov/Blast.cgi, accessed on 7 November 2025) was used to identify homologous sequences in test sequences and in human proteins. Options employed were blastp and ClusteredNR. Sequence identities were considered to be those where six or more contiguous sites are each occupied by the same amino acid. The choice of six or more continuous amino acids was considered as a compromise between accepting all the results suggested by NCBI BLAST and a more manageable number. Analysis was pursued only with proteins where the homologous region is at or near the C- or the N-terminus because these sites are expected to be exposed on the protein surface and able to interact with other components of the cytoplasm.

### 2.3. Other Methods

The cellular location of individual human proteins were those given by Gene Cards (https://www.genecards.org/), Human Protein Atlas (https://www.proteinatlas.org/) and DeepLoc2.1 (https://services.healthtech.dtu.dk/services/DeepLoc-2.1/, accessed on 7 November 2025). All three methods were used in the event of a disagreement in the tissue(s) identified. Peptide mass values were derived from ProteomicsDB (https://www.proteomicsdb.org/peptide, accessed on 7 November 2025). Peptide structures were predicated by AlphaFold Server (https://alphafoldserver.com/welcome, accessed on 7 November 2025). Test amino acid sequences were randomized with Sequence Manipulation Suite (https://www.bioinformatics.org/sms2/, accessed on 7 November 2025).

## 3. Results

### 3.1. Study Beginning with MLPRLVLNS

The overall project consisted of two parallel studies, each beginning with one of the two main test sequences, MLPRLVLNS and MLARLVSNS. In each case, BLAST was used to probe the human genome for amino acid sequences matching the test sequence. Proteins with matches of six contiguous amino acids or more were then further examined for the presence of the other two features expected of a CR sequence: (1) location of the candidate sequence at or near the C- or N-terminus of the matching protein, and (2) location of the protein in the cytoplasm. Proteins with all three properties are suggested to have cytoplasmic retention function.

Table 1 shows the results of the study carried out, beginning with MLPRLVLNS. Proteins with sequences matching MLPRLVLNS or regions of it are shown in the “protein” column lines 1–18. Three proteins, MAP3K5, TRPC1, and TTPA (lines 1–3), were found to have the other two properties described above and are therefore proposed as proteins with CR activity. While proteins in lines 4–18 have matches with the test sequence, all fail one or both of the other CR properties and are not included in the CR candidate group. BCLC7, for instance, has a good match with the test protein sequence, but the matching sequence is not found at an end of the protein, and the protein is found in the nucleus, not the cytoplasm (see line 4). While MYCBPAP has a good matching sequence located near a protein end (PRLVLNS; see line 15), the matching sequence is also found in CNOT6L, a protein present in the nucleus (line 16). Therefore, neither protein is included in the list of CR candidates.

A control experiment was performed with a randomized version of the test protein sequence (see line 19). BLAST analysis was performed with the randomized sequence in the expectation that it would yield fewer matches with human proteins and fewer of the other properties expected of candidate CR sequences. The expected result would help validate use of the test sequence to identify novel CR-active sequences. The outcome supported the expected result (Table 1, lines 20–22). While 18 human proteins had matches with the authentic test sequence, only 3 were observed with the randomized version. Also, none of the matches with the randomized sequence were found at the end of its home protein.

Support for the bioinformatic approach used here comes from a study in which CR activity was found to be conferred by a short amino acid sequence of the human protein ERK2 (MAPK3) [6]. The active sequence was found near the C-terminus of the target gene as required of candidate CR peptides in the analysis reported here (Table 1, line 23).

### 3.2. Study Beginning with MLARLVSNS

Results of the study beginning with MLARLVSNS are shown in Table 2. They show that four proteins, CCDC57, MYH1, TNFAIP3, and SLC11A1, have qualifying sequence matches near a protein end and are also found in the cytoplasm but not the nucleus. The four are therefore suggested for CR function. The remaining matches between MLARLVSNS and a human protein fail the other tests for inclusion in the CR group because the sequence is not located at a match protein end or the protein has evidence of localization in the nucleus (Table 2, lines 5–19). For instance, while the match of TTC22 with MLARLVSNS is found near the C-terminus, the protein is found in the nucleus (line 6). A similar situation is observed with PDZD7 (line 19).

The BLAST results with MLARLVSNS stand out because 14 of the 19 sequence matches with human proteins involve the same six-amino-acid sequence, RLVSNS (lines 5–19). This result was not expected as three other contiguous six-amino-acid sequences are found in the test peptide (i.e., ARLVSN, LARLVL, and MLARLV) and would have a chance of being found in human genes. It is suggested that the missing sequences may have an unknown toxicity or, alternatively, that RLVSNS-containing proteins could have additional properties that extend the abundance of RLVSNS.

As in the case of the MLPRLVLNS study, a control analysis was carried out with a randomized version of MLARLVSNS using the same BLAST procedure used for the unmodified sequence (Table 2, lines 20–24). The results with randomized MLARLVSNS yielded a lower number of matches with human genes than the unmodified sequence. Four matches were found with the randomized version compared with 19 in the original one (see the protein column in Table 2). Other features of the randomized sequence, such as location in the nucleus, disqualify the randomized version for inclusion in the candidate CR group.

### 3.3. Protein Isoform Analysis

Isoforms of proteins with candidate CR sequences were examined in case they would be found to have candidate CR sequences different from the original isoforms identified. Novel candidate CR sequences were found in isoforms of five of the proteins described above to have proposed CR signals: MAP3K5, TRPC1, TTPA, CCDC57, and MYH7 (Table 3 and Table 4). In each case, the CR sequence found in the new isoform was observed in the same location as that in the original peptide. This situation is illustrated in the case of MAP3K5 (Table 3). Here, the original test amino acid sequence, MLPRLVLNS, is found in two isoforms, X8 and X9, where it is located 18 amino acids away from the C-terminal end (see Table 3, lines 1 and 2). In agreement, the novel sequence DLKCLRLRG is found in isoforms 1, 2, and 6 where it is also located 18 amino acids away from the C-terminal end. This similarity is observed even though isoforms X8/X9 and 1/2/6 differ substantially in length (i.e., ~800 aa in X8/X9 vs. 1155–1465 aa in isoforms 1, 2, and 6; see Table 3 and Table 5). The same identity of isoform and proposed CR location was observed with isoforms of TTPA. Here, each of the three isoforms examined has a different candidate CR peptide, yet each is located at the C-terminus of the protein (Table 3, lines 9–11). The results are interpretated to indicate that the location of the CR sequence is the same in all isoforms of a particular protein, while isoforms may be the same or different in the sequence of their CR sequence.

The highest number of novel CR sequences was observed in CCDC57 (Table 4). Here, a total of five distinct proposed CR sequences is distributed among 13 isoforms. In each isoform, the proposed CR sequence is located at the same position in the CCDC57 protein beginning 5–6 amino acids away from the N-terminus. An unusual feature was observed in CCDC57 isoform X10 where two different, novel CR sequences were found at the N-terminal site usually occupied by only one. Here, both LCKKTMMCH and SEPALNELL are found between positions 5 and 34 (line 9).

### 3.4. Predicted Structures of Candidate CR Peptides

**Figure 1:**
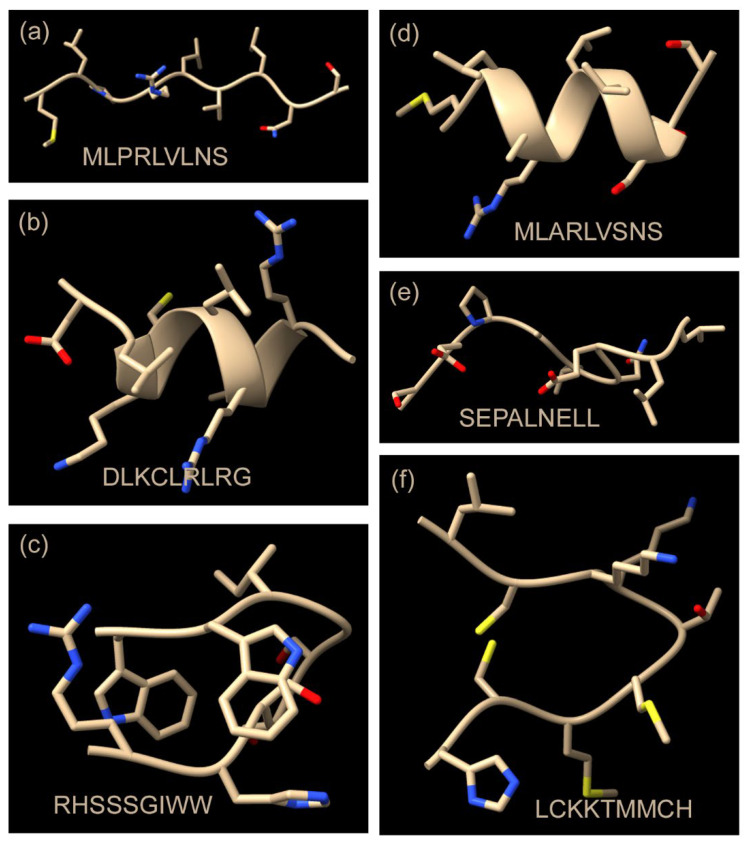
Predicted structures of selected candidate cytoplasmic retention sequences identified here. The structures can be described as “extended” in the case of MLPRLVLNS and SEPALNELL (**a**,**e**), “short α-alpha helix” in the case of MLARLVSNS and DLKCLRLRG (**b**,**d**), and “extended U shape” for RHSSSGIWW and LCKKTMMCH (**c**,**f**). Note that the predicted structures are diverse, as expected from their diverse amino acid sequences and their expected function in cytoplasmic retention.

The analysis described above yielded identification of 15 novel, candidate CR amino acid sequences (Table 6). The list is noteworthy for the amino acid sequence heterogeneity observed. Heterogeneity was observed in all but five of the candidate CR sequences identified here (Table 6, lines 1 and 7–10). Apart from the five sequences, however, it is difficult to identify sequences that have obvious regions of similarity. The observed sequence heterogeneity suggests a corresponding heterogeneity in peptide structure, and this feature was examined by using AlphaFold to predict the structure of each peptide. It was expected that the structures predicted by AlphaFold would confirm that the structures of the candidate CR sequences are indeed distinct. A sample of the results is shown in Figure 1.

Most of the structures could be assigned to one of three groups: (1) an extended array of amino acids as found in MLPRLVLNS and SEPALNELL; (2) a short α-helix (MLARLVSNS and DLKCLRLRG); and (3) a structure in which the peptide is folded into a U shape shown with the closed end shown at the right in RHSSSGIWW and LCKKTMMCH. In all three cases, the structures are interpreted to support the view that they are highly distinct from each other. This can be seen in the α-helical pair shown in Figure 1. While DLKCLRLRG is found to be rich in positively charged amino acids, MLARLVSNS is less so (compare panels (b) and (d) in Figure 1). DLKCLRLRG has regularly spaced leucine residues characteristic of α-helices [9]. In the extended chain pair, SEPALNELL is found to have a hydrophobic C-terminus not found in the other extended chain structure (compare panels (a) and (e)). In the LCKKTMMCH structure, two cysteine residues are near each other and potentially able to form a disulfide bond not seen in the other structures. The results of the predicted structures are interpreted to be consistent with the diverse sequences of the peptides examined and with the function of the sequences in the CR of the parent protein.

## 4. Discussion

The major contribution described here is a bioinformatic method to identify protein amino acid sequences involved in retaining their parent protein in the cytoplasm. In its environment in the cell, each sequence identified is expected to be a part of a donor–acceptor pair in which both proteins spend at least a part of their lives in the cytoplasm. As there are many such cytoplasmic proteins, it is expected that there are many amino acid sequences devoted to moving proteins to the right place in the cytoplasm (e.g., the mitochondria) and keeping them in place unless movement serves another requirement of the cell. The method described here is expected to provide researchers with a rapid process to identify candidate CR sequences by focusing on their basic properties, presence of the parent protein in the cytoplasm, and location of the CR sequence at an end of the protein amino acid sequence.

To validate the proposed method, it has been used to identify fifteen novel candidate CR sequences in human proteins. All have the properties expected of a CR sequence, including the observation that the group is quite diverse in an amino acid sequence, as would be expected of a small number of sequences selected from a much larger pool. It is hoped that the new method will enable further characterization of CR sequences, including information about the nature of the binding sites recognized by CR sequences.

In creating the new method for identification of CR sequences, a decision had to be made about what location(s) in the parent protein was to be accepted. The C-terminus and the N-terminus were both accepted as good experimental evidence supports the involvement of both [4,6,7,10,11,12,13,14]. However, it is acknowledged that sequences in the middle of a protein may also have cytoplasmic retention function. A disordered three-dimensional structure is one property found in sequences at protein ends, and disordered regions are also found in the middle of cytoplasmic proteins. Future studies of CR signals may therefore benefit from consideration of sequences in the middle of the parent protein.

Of the observations reported here, the most consequential may be those regarding isoforms of CR-containing proteins. It is observed that while different isoforms of a cytoplasmic protein may have CR sequences that are the same or different, the location of the CR sequence is the same in all isoforms. This was found to be the case even in the protein CCDC57 that has the most isoforms (13) and the most distinct CR sequences (5) of the proteins examined. Although other explanations may apply, we assume this arises by alternative splicing of the relevant gene followed by evolutionary adjustment of the isoform length so that the CR sequence can be accommodated by its binding site in the cytoplasm [15].

The presence of different CR sequences in the same isoform of a protein as described here could have important consequences for the overall organization of the cytoplasm. By using distinct CR sequences, the cell could direct the same protein isoform to distinct locations in the cytoplasm. For example, by using separate CR signals, an isoform of protein A could be directed to distinct locations in the cytoplasm such as lysosomes and the mitochondria. This would enable the cell to use its genes economically by distributing them to separate sites.

It is assumed that retention of a protein in the cytoplasm involves contact between its CR sequence and a protein receptor site in the cytoplasm. Protein–receptor contact is expected to be similar to those involved in other protein–protein interactions, except that one of the pair needs to be a resident of the cytoplasm such as a microtubule or a component of the endoplasmic reticulum membrane. In the future, it may be productive to examine the nature of the relevant interactions using bioinformatic software available for studying protein–protein interactions.

## 5. Conclusions

Three important, novel findings are reported here. (1) A total of 15 novel human cytoplasmic retention sequences are reported. All 15 are 7–9 amino acids in length, found in proteins present in the cytoplasm for at least a part of their lifetime in the cell, and located at or near the N- or the C-terminus of the protein (see Table 6). (2) A novel experimental pathway is described that allows researchers to identify additional cytoplasmic retention sequences. Using NCBI BLAST, test sequences are examined for homology with sequences in protein-coding genes of the human genome. Proteins with homologous regions of six or more contiguous amino acids are accepted as cytoplasmic retention sequences if they are also found in a cytoplasmic gene and located at or near a protein end. Since many cytoplasmic retention sequences are expected, researchers should be able to find many ways to employ the new method. (3) Among isoforms of the same protein, novel cytoplasmic retention sequences were found to be located at the same position in the amino acid sequence. This feature permits a protein isoform to be distributed to distinct locations in the cytoplasm.

## Figures and Tables

**Table 1:** Identification of human proteins with proposed cytoplasmic retention sequence MLPRLVLNS.

Line	Amino Acid Sequence	Protein	Isoform	Protein Length (aa)	Sequence Location in Protein	Protein Location in Cell
1	MLPRLVLNS	MAP3K5	X9	777	751–759	cytosol, ER
2	MLPRLVLNS	TRPC1	16	308	17–25	PM, cytosol, ER
3	MLPRLVLNS	TTPA	4	282	274–282	cytosol
						
4	LPRLVLNS	BCL7C	X1	324	183–190	nucleus
5	RLVLNS	SLC28A1	4	543	536–541	PM, cytosol
6	RLVLNS	KLRC3	E	240	235–240	PM, cytosol
7	MLPRLV	DUOXA1	1	483	434–439	ER, PM
8	MLPRLV	TTC2	2	372	346–351	cytosol, nucleus
9	RLVLNS	TRMT10B	X5	285	262–267	cytosol, nucleus
10	MLPRLV	GVQW3	d	187	161–166	cytosol, nucleus
11	MLPRLV	TERF2IP	CRA_b	161	1–6	nucleus, cytosol
12	MLPRLV	NEU3	e	139	108–113	lysosome
13	MLPRLV	ZNHIT1	CRA_b	129	1–6	nucleus, cytosol
14	MLPRLV	ETFA	2	45	19–24	mitochondria, golgi
15	PRLVLNS	MYCBPAP	CRA_a	855	33–39	endosome, PM
16	PRLVLNS	CNOT6L	5	587	299–305	cytosol, nucleus
17	RLVLNS	ALG8	8	557	191–196	ER, PM
18	MLPRLV	EIF4E	2	248	139–144	cytosol, nucleus
						
	Randomize sequence:					
19	LMLLPNVRS					
20	LLPNVR	ADAMYS9	4	1907	639–644	ER, PM
21	LPNVRS	CWF19L2	none	894	700–705	nucleus
22	LPNVRS	GK	d	559	230–235	cytosol, nucleus
						
	Rubinfeld et al. [6]					
23	YLEQYYDPS	MAPK3	1	379	329–336	golgi, cytosol

ER: endoplasmic reticulum; PM: plasma membrane.

**Table 2:** Identification of human proteins with proposed cytoplasmic retention sequence MLARLVSNS.

Line	Amino Acid Sequence	Protein	Isoform	Protein Length (aa)	Sequence Location in Protein	Protein Location in Cell
1	MLARLVSNS	CCDC57	X18	853	5–13	cytoskeleton
2	MLARLVLNS	MYH7	beta CRA_c	951	943–951	cytoskeleton, cytosol
3	ARLVSNS	TNFAIP3	CRA_c	353	326–332	cytosol
4	MLARLVSN	SLC11A1	11, 1, CRA_c	107	73–80	endosome, lysosome
						
5	RLVSNS	DUOXA1	1	483	437–442	ER, PM
6	RLVSNS	TTC22	2	372	349–354	cytosol, nucleus
7	RLVSNS	PDE6D	delta X1	164	4–9	cytosol, cytoskeleton
8	RLVSNS	NEU3	e	139	111–116	lysosome, endosome, PM
9	RLVSNS	MYL10	none	226	4–9	mitochondria, cytosol
10	RLVSNS	XPNPEP3	none	278	273–278	mitochondria, cytosol
11	RLVSNS	PTPN2	X1	494	50–55	cytosol, ER
12	RLVSNS	ITLN1	CRA_b	295	272–277	cytosol, PM
13	RLVSNS	HSPE1	CRA_g	124	95–100	mitochondria, cytosol
14	RLVSNS	TOM1	CRA_a	265	242–247	cytosol, endosome
15	RLVSNS	ARHGAP8	2	233	145–150	cytosol
16	RLVSNS	GVQW3	delta X1	187	164–169	cytosol, nucleus
17	RLVSNS	ZNHIT1	CRA_b	129	4–9	nucleus, cytosol
18	RLVSNS	ETFA	2	45	22–27	mitochondria
19	RLVSNS	PDZD7	3	539	4–9	nucleus, cytoskeleton
						
	Randomized sequence:					
20	LSSNVLAMR					
21	LSSNVL	STKLD1	none	680	176–180	cytosol, nucleus
22	LSSNVL	LNPK	1	494	282–287	ER, nucleus
23	SSNVLA	NEDD4	4	1303	102–107	golgi, cytosol, nucleus
24	SSNVLA	NAPRT	1	538	194–199	cytosol, nucleus

ER: endoplasmic reticulum; PM: plasma membrane.

**Table 3:** Proposed cytoplasmic retention sequences related to MLPRLVLNS in isoforms of human proteins.

Line	Protein	Amino Acid Sequence	Isoform	Protein Length (aa)	Amino Acid Location in Protein
1	MAP3K5	MLPRLVLNS	X9	777	751–759
2	MAP3K5	MLPRLVLNS	X8	813	787–795
3	MAP3K5	DLKCLRLRG	X6	1181	1155–1163
4	MAP3K5	DLKCLRLRG	1	1483	1457–1465
5	MAP3K5	DLKCLRLRG	2	1374	1348–1356
6	TRPC1	MLPRLVLNS	16	308	17–25
7	TRPC1	SSLPSSPSS	1	793	17–25
8	TRPC1	IQNPEYSTT	13	454	17–25
9	TTPA	MLPRLVLNS	4	282	274–282
10	TTPA	LSSISESIQ	6	317	309–317
11	TTPA	RHSSSGIWW	3	145	137–145

**Table 4:** Proposed cytoplasmic retention sequences related to MLARLNSNS in isoforms of human proteins.

Line	Protein	Amino Acid Sequence	Isoform	Protein Length (aa)	aa Location in Protein
1	CCDC57	MLARLVSNS	X18	853	5–13
2	CCDC57	SEPALNELL	4	1027	6–14
3	CCDC57	SEPALNELL	1	915	6–14
4	CCDC57	SEPALNELL	X7	1106	5–13
5	CCDC57	LCKKTMMCH	X25	528	5–13
6	CCDC57	LCKKTMMCH	X24	530	5–13
7	CCDC57	LCKKTMMCH	X23	650	5–13
8	CCDC57	LCKKTMMCH	X19	850	5–13
9	CCDC57	LCKKTMMCH plus	X10	1047	5–34
		SEPALNELL			
10	CCDC57	TLRQSVEEV	X3, X6, X8	1117	5–13
11	CCDC57	EHEFRLQAD	X13	925	5–13
12	MYH7	MLARLVLNS	CRA_c	951	943–951
13	MYH7	MLARLVLNS	CRA_a	1945	1937–1945
14	MYH7	IGTKGLNEE	CRA_b	1935	1927–1935

**Table 5:** Isoform and CR sequence location in human MAP3K5.

Protein	CR Seq	Isoform	Start	Stop	Protein	aa Sequence	C-Terminus
MAP3K5	MLPRLVLNS	X9	742	777	LLERLVLV	**MLPRLVLNS**	WAQAILPPRPPKALELQA
MAP3K5	MLPRLVLNS	X8	778	813	LLERLVLV	**MLPRLVLNS**	WAQAILPPRPPKALELQA
MAP3K5	DLKCLRLRG	X6	1146	1181	VLYYVTRD	**DLKCLRLRG**	GMLCTLWKAIIDFRNKQT
MAP3K5	DLKCLRLRG	1	1448	1483	VLYYVTRD	**DLKCLRLRG**	GMLCTLWKAIIDFRNKQT
MAP3K5	DLKCLRLRG	2	1339	1374	VLYYVTRD	**DLKCLRLRG**	GMLCTLWKAIIDFRNKQT

**Table 6:** Candidate CR peptides reported here.

No.	Peptide Sequence
1	MLPRLVLNS
2	DLKCLRLRG
3	SSLPSSPSS
4	IQNPEYSTT
5	LSSISESIQ
6	RHSSSGIWW
7	MLARLVSNS
8	MLARLVLNS
9	ARLVSNS
10	MLARLVSN
11	SEPALNELL
12	LCKKTMMCH
13	TLRQSVEEV
14	EHEFRLQAD
15	IGTKGLNEE

## Data Availability

The data presented in this study are available in the article itself.
